# Differential Carbohydrate Recognition by *Campylobacter jejuni* Strain 11168: Influences of Temperature and Growth Conditions

**DOI:** 10.1371/journal.pone.0004927

**Published:** 2009-03-17

**Authors:** Christopher J. Day, Joe Tiralongo, Regan D. Hartnell, Carie-Anne Logue, Jennifer C. Wilson, Mark von Itzstein, Victoria Korolik

**Affiliations:** Institute for Glycomics, Griffith University Gold Coast Campus, Queensland, Australia; Charité, Campus Benjamin Franklin, Germany

## Abstract

The pathogenic clinical strain NCTC11168 was the first *Campylobacter jejuni* strain to be sequenced and has been a widely used laboratory model for studying *C. jejuni* pathogenesis. However, continuous passaging of *C. jejuni* NCTC11168 has been shown to dramatically affect its colonisation potential. Glycan array analysis was performed on *C. jejuni* NCTC11168 using the frequently passaged, non-colonising, genome sequenced (11168-GS) and the infrequently passaged, original, virulent (11168-O) isolates grown or maintained under various conditions. Glycan structures recognised and bound by *C. jejuni* included terminal mannose, *N*-acetylneuraminic acid, galactose and fucose. Significantly, it was found that only when challenged with normal oxygen at room temperature did 11168-O consistently bind to sialic acid or terminal mannose structures, while 11168-GS bound these structures regardless of growth/maintenance conditions. Further, binding of un-capped galactose and fucosylated structures was significantly reduced when *C. jejuni* was maintained at 25°C under atmospheric oxygen conditions. These binding differences identified through glycan array analysis were confirmed by the ability of specific lectins to competitively inhibit the adherence of *C. jejuni* to a Caco-2 intestinal cell line. Our data suggests that the binding of mannose and/or *N*-acetylneuraminic acid may provide the initial interactions important for colonisation following environmental exposure.

## Introduction

Carbohydrates (or glycans) that modify proteins and lipids play a key role in numerous cell recognition events, including those involved in the regulation of the immune system [1], and in the attachment of pathogenic organisms to host tissue [2]. Protein-carbohydrate interactions have been identified as adherence factors for numerous commensal and pathogenic bacteria including *Pseudomonas aeruginosa* [3], [4], [5], *Helicobacter pylori* [6], [7], [8], [9], [10] and *Escherichia coli* [11]. The identification and characterisation of carbohydrate binding proteins (or lectins) has been greatly enhanced through the development of glycan array technology [12]. Glycan arrays are fast becoming the technique of choice for identifying and elucidating the specificity of lectins [12], [13], [14], and have been successfully used to identify and characterise the interactions of viruses and bacteria with their glycan receptors [12], [15].

*Campylobacter jejuni*, the most prevalent cause of gastroenteritis in developed countries, is a highly motile, Gram-negative spiral rod that requires microaerobic conditions for growth [16], [17]. *C. jejuni* is a zoonotic pathogen, being a commensal organism in poultry and other wildlife. Poorly prepared poultry products represent the most common source of human infection; however, infection can also arise from contaminated water and unpasteurised milk [16]. *C. jejuni* is a thermophilic organism, requiring temperatures in the range of 32°C to 45°C for growth, but it can also survive at lower temperatures in the environment [18].

The pathogenic clinical isolate NCTC11168 was the first *C. jejuni* strain to be sequenced and has been a widely used laboratory model for studying *C. jejuni* pathogenesis [19]. However, more recent investigations have shown that continuous passaging of *C. jejuni* NCTC11168 dramatically affects its colonisation potential. That is, long-term passaging and oxygen-adaptation of the 11168-genome sequenced strain (11168-GS) leads to poor colonisation potential in chicken models [20], [21], while the same strain infrequently passaged (11168-original, 11168-O) remains a potent coloniser of chickens. Moreover, *C. jejuni* 11168-O was found to be more adherent and invasive in *in vitro* infection models and was observed to be more motile than 11168-GS [21]. Microscopic analysis of the two strains also demonstrated a shift in phenotypic appearance, with 11168-GS existing as straight rods rather than the normal spiral-shaped rod of 11168-O. Other differences were also noted in gene expression, with particular changes in genes involved in oxygen metabolism; however, serotyping, genotyping and subtractive hybridisation found the two strains to be clonal [21].

*C. jejuni* does not ferment or oxidise carbohydrates as a carbon source, instead relying on amino acids such as aspartate and serine [22]. Any observed carbohydrate binding can therefore be attributed to interactions important for adherence and/or colonisation rather than for energy acquisition. The differential adherence, invasion and colonisation observed for *C. jejuni* 11168-GS and 11168-O suggests that significant differences exist in how the two strains interact with host cells. Bacteria-host interactions depend not only on bacterial motility, but also on bacterial adherence factors such as lectins, and the surface composition of the host cell, including the degree and type of glycosylation [2], [23]. The adherence phenotypes observed for *C. jejuni* 11168-GS and 11168-O may therefore reflect differential host cell carbohydrate recognition. The carbohydrate-mediated interaction of *C. jejuni* with host cell glycans has been reported. *C. jejuni*, for example, is chemotactic to fucose (Fuc) [24], and is known to interact with glycoproteins of the intestinal tract, such as mucins [25] and Lewis B-like structures present on human milk proteins [26], [27]. The functional significance of these interactions is not entirely understood, and the complete range of sugars recognised, as well as the *C. jejuni* lectins involved, are yet to fully be identified.

This study aims to identify glycan structures bound by *C. jejuni* 11168-GS and 11168-O strains using glycan array technology and *in vitro* cell adherence assays, and to explore how these interactions are influenced by changes in temperature and oxygen condition. Conditions were chosen to mimic those encountered by *C. jejuni* through various growth and survival phases, including conditions required for survival in the environment (25°C, normal O_2_), and in mammalian (37°C, microaerophilic) and avian (42°C, microaerophilic) hosts (Table 1). Here we report that distinct growth and survival conditions dramatically alter the degree and specificity of *C. jejuni* binding to various carbohydrate structures, including differential binding to mannose (Man) and *N*-acetylneuraminic acid (Neu5Ac)-containing glycans.

**Table 1:** *C. jejuni* 11168 genome sequenced (11168-GS) and 11168 original (11168-O) growth and/or maintenance conditions investigated.

	Temperature	Atmosphere	Growth/maintenance time (hrs)
Environment			
11168-GS 25°C	20–25°C	Normal	24
11168-O 25°C	20–25°C	Normal	24
Mammalian			
11168-GS 37°C	37°C	Microaerobic	36
11168-O 37°C	37°C	Microaerobic	36
Avian			
11168-GS 42°C	42°C	Microaerobic	20
11168-O 42°C	42°C	Microaerobic	20

## Results

### Glycan array analysis

Glycan arrays were performed using the non-colonising genome sequenced isolate of the *C. jejuni* strain NCTC11168 (11168-GS) and the colonising isolate 11168-O, grown or maintained at varying conditions as outlined in the Materials and Methods (summarised in Table 1). Our analyses found that the binding of *C. jejuni* strains 11168-GS and 11168-O to galactose (Gal), Man, Fuc, and Neu5Ac-containing glycans was significantly affected by the conditions under which the bacteria were grown/maintained. In addition, significant differences in the carbohydrate-binding specificity were also observed between *C. jejuni* 11168-GS and 11168-O.

### Binding to terminal Gal/GalNAc

**Figure 1:**
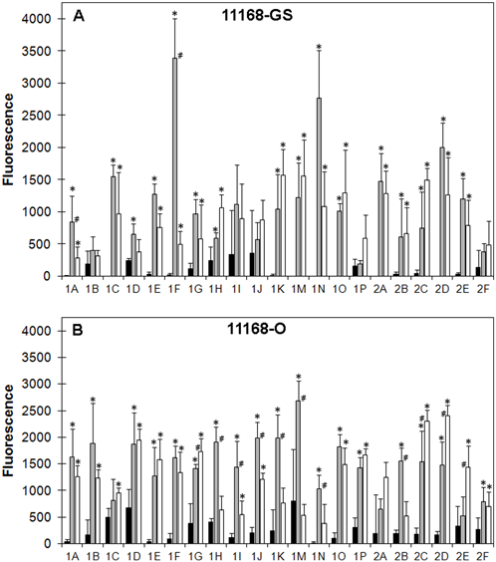
Binding of *C. jejuni* 11168 to uncapped terminal Gal structures. Fluorescence intensities associated with *C. jejuni* 11168-GS (A) and *C. jejuni* 11168-O (B) binding to uncapped Gal structures (25°C, black bar; 37°C, grey bar; and 42°C, white bar). For the structure of the individual glycans refer to Table 2. * Significantly different to 25°C, P<0.05; # Significant difference between 37°C and 42°C, P<0.05.

*C. jejuni* 11168-GS maintained at 25°C (11168-GS 25°C) exhibited little or no binding to terminal Gal-containing structures present on the array. Of particular note, no binding to the core O-glycan Tn antigen (Galβ1-3GalNAcα1-O-Ser; 1M) or glycans terminated with Galα1-3/4Gal (1K, 1N, 1O, 1P and 2A; see table 2 for structures) was observed (Figure 1A). The growth of 11168-GS at 37°C under a microaerobic atmosphere, which mimics mammalian host-like conditions (11168-GS 37°C), and at 42°C under a microaerobic atmosphere, which mimics avian host-like conditions (11168-GS 42°C), resulted in significantly greater binding (P<0.05) to terminal Gal structures in comparison to 11168-GS 25°C. In fact, *C. jejuni* 11168-GS 37°C and 11168-GS 42°C bound all terminal Gal structures present on the array (Figure 1A). However, structures such as Galβ1-3GlcNAc, Galβ1-3GalNAc, Galβ1-3GalNAcβ1-4Galβ1-4Glc and Galα1-3Gal (1A, E, F and N respectively; Figure 1 and Table 2) were bound significantly less by 11168-GS 42°C in comparison to 11168-GS 37°C. Conversely, the structure Galβ1-4GlcNAcβ1-3Galβ1-4Glc (1H, Figure 1 and Table 2) was bound significantly more by 11168-GS 42°C than 11168-GS 37°C. Even though some differences in the level of binding to various terminal Gal structures by *C. jejuni* 11168-GS grown at conditions that mimic mammalian and avian hosts were noted, there appeared to be no significant specificity requirement for a particular glycosidic linkage or underlying sugar.

**Table 2:** Glycan structures present on array.

ID	Glycan structure
1A	Galβ1-3GlcNAc
1B	Galβ1-4GlcNAc
1C	Galβ1-4Gal
1D	Galβ1-6GlcNAc
1E	Galβ1-3GalNAc
1F	Galβ1-3GalNAcβ1-4Galβ1-4Glc
1G	Galβ1-3GlcNAcβ1-3Galβ1-4Glc
1H	Galβ1-4GlcNAcβ1-3Galβ1-4Glc
1I	Galβ1-4GlcNAcβ1-6(Galβ1-4GlcNAcβ1-3)Galβ1-4Glc
1J	Galβ1-4GlcNAcβ1-6(Galβ1-3GlcNAcβ1-3)Galβ1-4Glc
1K	Galα1-4Galβ1-4Glc
1L	GalNAcα1-O-Ser
1M	Galβ1-3GalNAcα1-O-Ser
1N	Galα1-3Gal
1O	Galα1-3Galβ1-4GlcNAc
1P	Galα1-3Galβ1-4Glc
2A	Galα1-3Galβ1-4Galα1-3Gal
2B	Galβ1-6Gal
2C	GalNAcβ1-3Gal
2D	GalNAcβ1-4Gal
2E	Galα1-4Galβ1-4GlcNAc
2F	GalNAcα1-3Galβ1-4Glc
2G	Galβ1-4GlcNAcβ1-6(Galβ1-4GlcNAcβ1-3)Galβ1-4Glc
2H	Galβ1-3GlcNAcβ1-3Galβ1-4GlcNAcβ1-6(Galβ1-3GlcNAcβ1-3)Galβ1-4Glc
2I	Galβ1-3GlcNAcβ1-3Galβ1-4GlcNAcβ1-3Galβ1-4Glc
4A	GlcNAcβ1-4GlcNAc
4B	GlcNAcβ1-4GlcNAcβ1-4GlcNAc
4C	GlcNAcβ1-4GlcNAcβ1-4GlcNAcβ1-4GlcNAc
4D	GlcNAcβ1-4GlcNAcβ1-4GlcNAcβ1-4GlcNAcβ1-4GlcNAcβ1-4GlcNAc
4E	GlcNAcβ1-4MurNAc
5A	GlcNAcβ1-2Man
5B	GlcNAcβ1-2Manα1-6(GlcNAcβ1-2Manα1-3)Man
5C	Manα1-2Man
5D	Manα1-3Man
5E	Manα1-4Man
5F	Manα1-6Man
5G	Manα1-6(Manα1-3)Man
5H	Manα1-6(Manα1-3)Manα1-6(Manα1-3)Man
7A	Fucα1-2Galβ1-3GlcNAcβ1-3Galβ1-4Glc
7B	Galβ1-3(Fucα1-4)GlcNAcβ1-3Galβ1-4Glc
7C	Galβ1-4(Fucα1-3)GlcNAcβ1-3Galβ1-4Glc
7D	Fucα1-2Galβ1-3(Fucα1-4)GlcNAcβ1-3Galβ1-4Glc
7E	Galβ1-3(Fucα1-4)GlcNAcβ1-3Galβ1-4(Fucα1-3)Glc
7F	Fucα1-2Gal
7G	Fucα1-2Galb1-4Glc
7H	Galβ1-4(Fucα1-3)Glc
7I	Galβ1-4(Fucα1-3)GlcNAc
7J	Galβ1-3(Fucα1-4)GlcNAc
7K	GalNAcα1-3(Fucα1-2)Gal
7L	Fucα1-2Galβ1-4(Fucα1-3)Glc
7M	Galβ1-3(Fucα1-2)Gal
7N	Fucα1-2Galβ1-4(Fucα1-3)GlcNAc
7O	Fucα1-2Galβ1-3GlcNAc
7P	Fucα1-2Galβ1-3(Fucα1-4)GlcNAc
8A	SO_3_-3Galβ1-3(Fucα1-4)GlcNAc
8B	SO_3_-3Galβ1-4(Fucα1-3)GlcNAc
8C	Galβ1-3GlcNAcβ1-3Galβ1-4(Fucα1-3)GlcNAcβ1-3Galβ1-4Glc
8D	Galβ1-4(Fucα1-3)GlcNAcβ1-6(Galβ1-3GlcNAcβ1-3)Galβ1-4Glc
8E	Galβ1-4(Fucα1-3)GlcNAcβ1-6(Fucα1-2Galβ1-3GlcNAcβ1-3)Galβ1-4Glc
8F	Galβ1-4(Fucα1-3)GlcNAcβ1-6(Fucα1-2Galβ1-3(Fucα1-4)GlcNAcβ1-3)Galβ1-4Glc
10A	Neu5Acα2-3Galβ1-3(Fucα1-4)GlcNAc
10B	Neu5Acα2-3Galβ1-4(Fucα1-3)GlcNAc
10C	Neu5Acα2-3Galβ1-3GlcNAcβ1-3Galb1-4Glc
10D	Galβ1-4(Fucα1-3)GlcNAcβ1-6(Neu5Acα2-6Galβ1-4GlcNAcβ1-3)Galβ1-4Glc
10E	Neu5Acα2-3Galβ1-3(Neu5Acα2-6)GalNAc
10F	Fucα1-2Galβ1-3(Neu5Acα2-6)GlcNAcβ1-3Galβ1-4Glc
10G	Neu5Acα2-3Galβ1-3(Fucα1-4)GlcNAcβ1-3Galβ1-4Glc
10H	Neu5Acα2-6Galβ1-3GlcNAcβ1-3Galβ1-4(Fucα1-3)Glc
10I	Neu5Acα2-6Galβ1-4GlcNAcβ1-6(Galβ1-3GlcNAcβ1-3)Galβ1-4Glc
10J	Galβ1-4GlcNAcβ1-6(Neu5Acα2-6Galβ1-4GlcNAcβ1-3)Galβ1-4Glc
10K	Neu5Acα2-3Galβ1-4GlcNAc
10L	Neu5Acα2-6Galβ1-4GlcNAc
10M	Neu5Acα2-3Galβ1-3GlcNAcβ1-3Galβ1-4Glc
10N	Galβ1-3(Neu5Acα2-6)GlcNAcβ1-3Galβ1-4Glc
10O	Neu5Acα2-6Galβ1-4GlcNAcβ1-3Galβ1-4Glc
10P	Neu5Acα2-3Galβ1-3(Neu5Acα2-6)GlcNAcβ1-3Galβ1-4Glc
11A	Neu5Acα2-3Galβ1-4Glc
11B	Neu5Acα2-6Galβ1-4Glc
11C	(Neu5Acα2-8Neu5Ac)_n_

With respect to Gal terminated glycans, binding of *C. jejuni* 11168-O (Figure 1B) had few statistically significant differences to the binding observed for 11168-GS (Figure 1A). For example the ability of *C. jejuni* 11168-O to bind terminal Gal structures generally increased as the growth temperatures increased (P<0.05). Specific differences however were observed between the two 11168 isolates grown at 37°C and 42°C. For example, 11168-O 37°C bound glycans 1H, 1I, 1J, 1K and 1M significantly more than 11168-O 42°C (P<0.05; Figure 1B). This is different than binding observed to the same glycans by 11168-GS grown at 37°C and 42°C, where no significant differences were observed (Figure 1A). Analogous to that observed for 11168-GS binding to terminal Gal structures, even though some differences in the level of binding to various terminal Gal structures by *C. jejuni* 11168-O grown under mammalian and avian-like conditions were noted, no significant differences in the specificity for a particular glycosidic linkage or underlying sugar could be deduced. As with 11168-GS, 11168-O grown at 37°C and 42°C, but not at 25°C had significant binding to terminal Galα1-3/4Gal structures (Figure 1B).

### Binding to Mannose containing glycans

**Figure 2 pone-0004927-g002:**
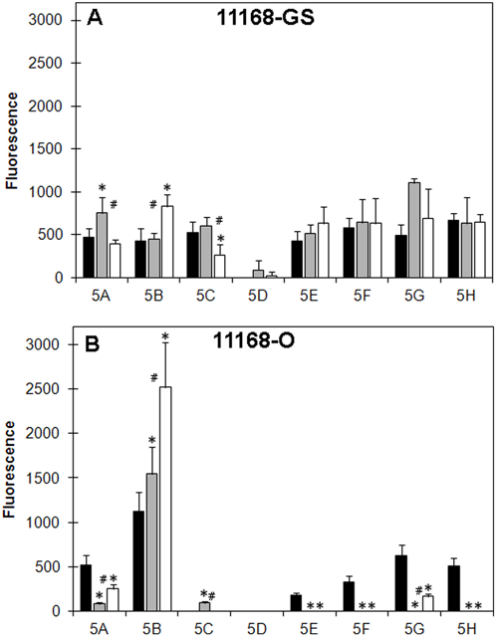
Binding of *C. jejuni* 11168 to Man containing structures. Fluorescence intensities associated with *C. jejuni* 11168-GS (A) and *C. jejuni* 11168-O (B) binding to Man containing glycans (25°C, black bar; 37°C, grey bar; and 42°C, white bar). For the structure of the individual glycans refer to Table 2. * Significantly different to 25°C, P<0.05; # Significant difference between 37°C and 42°C, P<0.05.

With respect to the ability of *C. jejuni* 11168 to bind Man-containing structures, figure 2A shows that *C. jejuni* 11168-GS bound multiple Man structures at low but detectable levels irrespective of the growth conditions tested (Figure 2A). One exception was the binding of 11168-GS to α1-3-mannobiose (Manα1-3Man; 5D), which was noticeably reduced. Interestingly, there were no significant differences observed in the level and specificity of *C. jejuni* 11168-O 25°C and 11168-GS 25°C binding to Man. However, 11168-O grown at conditions mimicking mammalian and avian hosts had, in the majority of cases, significantly reduced Man-binding in comparison to 11168-GS grown under the same conditions. The only exception to this was 11168-O binding to glycans containing GlcNAcβ (1-2)Man, particularly GlcNAcβ1-2Manα1-6(GlcNAcβ1-2Manα1-3))Man (5B), an important *N*-glycan biantennary core structure, which significantly increased as the growth temperature was increased, and to a lesser extent 5A (Figure 2B). Considered together, the data suggests that a distinct difference in Man-binding potential exists between *C. jejuni* 11168-GS and 11168-O when grown at conditions that are associated with either the mammalian or avian hosts.

### Binding to fucoslylated glycans

**Figure 3 pone-0004927-g003:**
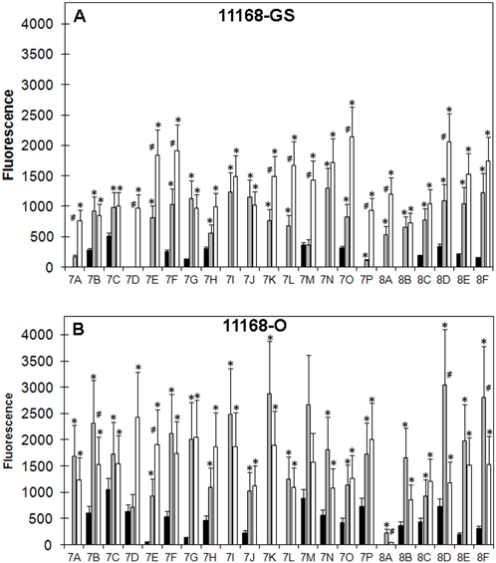
Binding of *C. jejuni* 11168 to fucosylated structures. Fluorescence intensities associated with *C. jejuni* 11168-GS (A) and *C. jejuni* 11168-O (B) binding to fucosylated glycans (25°C, black bar; 37°C, grey bar; and 42°C, white bar). For the structure of the individual glycans refer to Table 2. * Significantly different to 25°C, P<0.05; # Significant difference between 37°C and 42°C, P<0.05.

*C. jejuni* 11168 showed significant binding to a range of fucosylated glycans. *C. jejuni* 11168-GS maintained at 25°C, bound only approximately 50% of the fucosylated glycans present on the array (Figure 3A). Of particular note was the fact that no binding was observed for 11168-GS 25°C to the trisaccharides Lewis^a^ (Galβ1-3(Fucα1-4)GlcNAc; Le^a^; 7J) and Lewis^x^ (Galβ1-4(Fucα1-3)GlcNAc; Le^x^; 7I) (Figure 3A), even though detectable binding to other glycans terminated with Le^a^ and Le^x^ was observed (e.g. 7B and 7C, respectively). Further analysis found that 11168-GS 25°C bound monofucosylated glycans with a disaccharide backbone (7F, 7G, 7H, 7I, 7J, 7K, 7M, 7O, 8A and 8B) to a lesser extent than monofucosylated tetrasaccharide or larger glycans (7A, 7B, 7C, 8C and 8D; P = 0.04). As previously observed for 11168-GS binding to terminal Gal (Figure 1A), 11168-GS binding to Fuc was also dependent on the growth/maintenance conditions. That is, fucosylated structures were bound by 11168-GS 37°C and 11168-GS 42°C to a significantly greater extent than 11168-GS 25°C (Figure 3A).

Even though in comparison to 11168-GS 25°C a larger subset of fucosylated glycans was bound, *C. jejuni* 11168-O 25°C like 11168-GS 25°C was unable to recognise Le^a^ and Le^x^ (7J and 7I, respectively; Figure 3B). As was seen for 11168-GS the binding of 11168-O grown at 37°C and 42°C was significantly higher (P<0.05) than that seen for 11168-O 25°C (Figure 3B). However, unlike 11168-GS, there was no increase in 11168-O Fuc-binding as growth conditions were changed from 37°C microaerobic to 42°C microaerobic (Figure 3B). One of the few fucosylated structures that was poorly bound by 11168-O was sulfo-Le^a^ (8A, Figure 3B), which exhibited significantly reduced binding compared to 11168-GS grown under host-like conditions. These data highlight distinct differences in Fuc binding that is dependent both on the *C. jejuni* isolate (11168-GS or 11168-O), and the condition used to grow/maintain the bacteria.

### Binding to sialylated glycans

**Figure 4:**
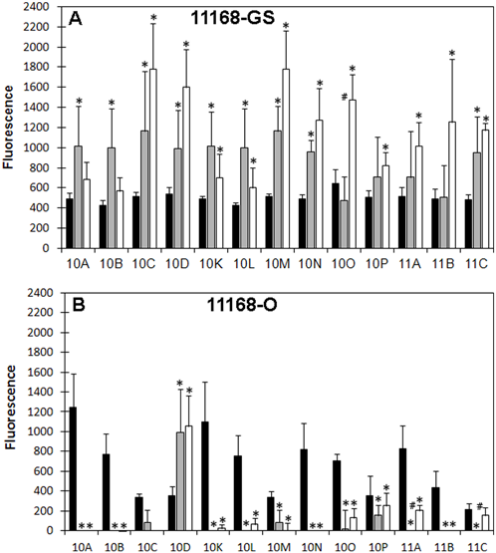
Binding of *C. jejuni* 11168 to Neu5Ac containing structures. Fluorescence intensities associated with *C. jejuni* 11168-GS (A) and *C. jejuni* 11168-O (B) binding to Neu5Ac containing glycans (25°C, black bar; 37°C, grey bar; and 42°C, white bar). For the structure of the individual glycans refer to Table 2. * Significantly different to 25°C, P<0.05; # Significant difference between 37°C and 42°C, P<0.05.

Our glycan array analyses also identified significant differences in the ability of *C. jejuni* 11168 to bind to Neu5Ac-containing glycoconjugates, with Neu5Ac-binding being affected not only by the growth/maintenance conditions, but also the isolate (11168-GS or 11168-O). Overall, *C. jejuni* 11168-GS bound all Neu5Ac-containing glycans present on the array (Figure 4A); however, increased binding was observed for 11168-GS 37°C and 11168-GS 42°C in comparison to 11168-GS 25°C (P<0.05; Figure 4A). *C. jejuni* 11168-O maintained at 25°C was also able to recognise and bind all sialylated glycans. Significantly however, Neu5Ac-binding was almost completely abolished when *C. jejuni* 11168-O was grown at 37°C and 42°C (Figure 4B). The only exception to this was 11168-O binding to monosialyl, monofucosyllacto-*N*-neohexaose (Galβ1-4(Fucα1-3)GlcNAcβ1-6(Neu5Acα2-6Galβ1-4GlcNAcβ1-3)Galβ1-4Glc; 10D; Figure 4B and Table 2), a fucosylated and sialylated biantennary glycan, which was significantly higher for 11168-O 37°C and 42°C in comparison to 11168-O 25°C (Figure 4B). It is interesting to note that binding to sialylated structures was independent of the Neu5Ac linkage (α2-3, α2-6 or α2-8) and the nature of the sub-terminal sugar for both 11168-GS and 11168-O (Figure 4). Taken together, these data indicate that significant differences exist between *C. jejuni* 11168-GS and 11168-O recognition of sialylated glycans when grown at conditions that are associated with either the mammalian or avian hosts.

**Figure 5:**
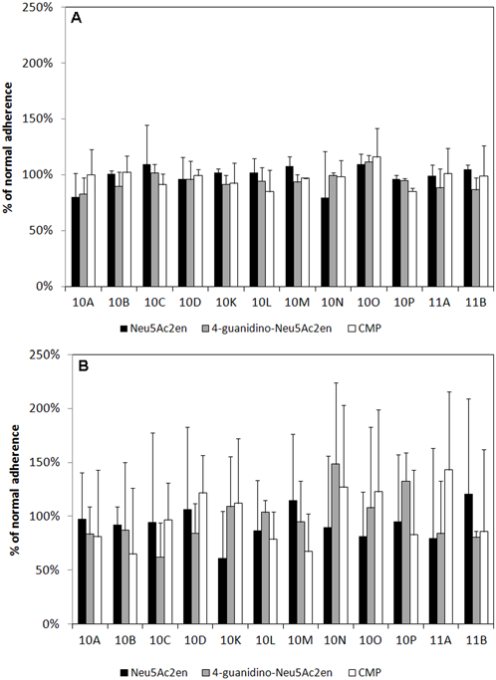
Relative binding of *C. jejuni* 11168 to Neu5Ac containing structures in the presence of sialyltransferase and sialidase inhibitors. Relative fluorescent levels of treated 11168-GS 42°C (A) and *C. jejuni* 11168-O 25°C (B) compared to normal *C. jejuni* binding to Neu5Ac containing glycans. (Neu5Ac2en, black bar; 4-guanidino-Neu5Ac2en, grey bar; and CMP, white bar). For the structure of the individual glycans refer to Table 2.

Recently, a sialidase activity was observed associated with the *C. jejuni* CSTII sialyltransferase following its expression in *E. coli* [28]. Given the differences observed in Neu5Ac-binding between 11168-GS (significant Neu5Ac binding) and 11168-O (little or no Neu5Ac binding), and that such sialidase activity could release terminal Neu5Ac from sialylated glycans on the glycan array, additional array analyses were performed in the presence of the sialyltransferase inhibitor CMP, and the sialidase inhibitors Neu5Ac2en and 4-guanidino-Neu5Ac2en (Zanamivir). Representative experiments were performed using 11168-O 25°C and 11168-GS 42°C. These conditions were chosen to allow for testing of both strains at their respective maximum observed Neu5Ac binding capacity. The analyses showed that the addition of these known inhibitors did not significantly alter the ability of 11168-GS 42°C and 11168-O 25°C to bind Neu5Ac containing glycans (P>0.52, Figure 5A and B). In fact, the addition of sialyltransferase and sialidase inhibitors had no effect on any of the glycan binding profiles shown in figures 1–4 for both *C. jejuni* 11168-GS 42°C and 11168-O 25°C (P>0.18).

### *In vitro* Caco-2 cell adherence assays

The specificity of glycan-mediated adherence of *C. jejuni* 11168 isolates were verified by *in vitro* Caco-2 binding assays following pre-treatment of cells with a competing lectin. The lectins selected were Erythrina cristagalli agglutinin (ECA; Galβ1-4Glc/GlcNAc>Gal), concanavalin A (ConA; Man), Ulex europaeus agglutinin 1 (UEA-1; terminal α1-2 Fuc), Limax flavus agglutinin (LFA; terminal sialic acid), Maackia amurensis agglutinin (MAA; Neu5Acα2-3>Gal) and Sambucus nigra agglutinin (SNA;Neu5Acα2-6). The effect of pre-treating Caco-2 cells with selected lectins on *C. jejuni* 11168 adherence was determined relative to binding observed in the absence of lectin, which was set at 100%. As previously observed by Gaynor *et al.*, (2004), we found that *C. jejuni* 11168-O (1.2% of inoculated bacteria) adhered to Caco-2 cells to a greater extent than 11168-GS (0.41% of inoculated bacteria) (P = 0.004) under standard assay conditions (see Materials and Methods).

**Figure 6:**
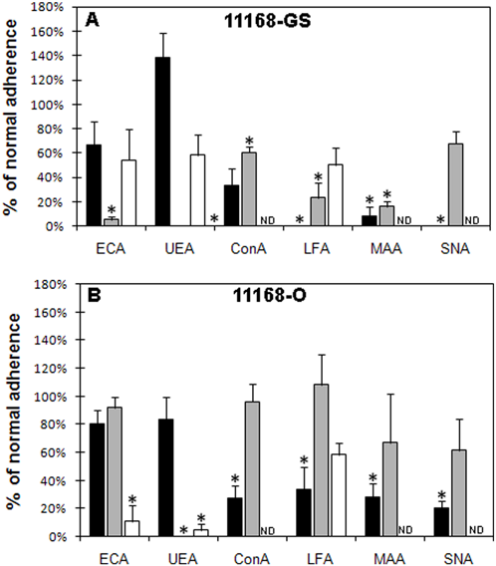
Lectin inhibition of *C. jejuni* adherence to intestinal cell line. *C. jejuni* 11168-GS (A) and 11168-O (B) adherence to Caco-2 cells pre-treated with ECA, UEA-1, ConA, LFA, MAA and SNA (25°C, black bar; 37°C, grey bar; and 42°C, white bar). * Significant difference to non-lectin treated control, P<0.05; ND, Not determined.

We found that treatment with ECA reduced *C. jejuni* 11168 adherence to terminal lactose/Gal structures present on the surface of Caco-2 cells. *C. jejuni* 11168-GS 37°C adherence to Caco-2 cells pre-treated with ECA was reduced by greater than 85% (P = 0.02). No statistically significant difference was observed for 11168-GS 25°C and 11168-GS 42°C (P>0.05); although, reductions of greater than 30% were observed (Figure 6A). *C. jejuni* 11168-O 42°C binding to Caco-2 cells was also significantly affected by ECA pre-treatment with a reduction of adherence of more than 80% observed (P = 0.04, Figure 6B). In contrast, the adherence of 11168-O 25°C and 37°C to pre-treated Caco2 cells was reduced by less than 10% (Figure 6B).

The affect of *C. jejuni* adherence to fucosylated structures on Caco-2 cells was determined using UEA-1. While no significant inhibition of adherence to UEA-1 pre-treated cells was observed for 11168-GS and 11168-O at 25°C (P>0.22, Figure 6A and B); 40–100% reduction in adherence of 11168-GS and 11168-O grown at both 37°C and 42°C was observed (P<0.05, Figure 6A and B).

Pre-treatment of Caco-2 cells with ConA resulted in greater than 40% reduction in 11168-GS and 11168-O 25°C (P = 0.02 and 0.045, respectively), and 11168-GS 37°C (P = 0.01) adherence (Figure 6A). However, adherence to Caco-2 cells by 11168-O 37°C was not affected (P = 0.96) by ConA pre-treatment (Figure 6B).

Initial cell binding assays using LFA to neutralise all terminal Neu5Ac residues present on Caco-2 cells were performed to evaluate the involvement of Neu5Ac on *in vitro* adherence. Figure 6A shows that a significant decrease in the adherence to Caco-2 cells of 11168-GS 25°C (P = 0.02) and 11168-GS 37°C (P = 0.04) was observed, with a 75–100% reduction in comparison to non-treated cells. A reduction of 50% was also observed for 11168-GS 42°C adherence; however, this was not statistically significant (P = 0.09, Figure 6A). Based on these results the adherence of 11168-GS to Caco-2 cells was further assessed by pre-treating cells with other sialolectins, MAA and SNA (Neu5Acα2-3Gal>Gal and Neu5Acα2-6Gal-specific lectins respectively). Both MAA and SNA pre-treatment resulted in significantly reduced adherence of 11168-GS 25°C (P = 0.01 and 0.02, respectively) to Caco-2 cells (Figure 6C). However, only MAA pre-treatment significantly reduced the adherence of 11168-GS 37°C to Caco-2 cells (Figure 6A), suggesting that 11168-GS 37°C recognition of Neu5Ac on Caco-2 cells is α2-3 specific.

As expected, pre-treatment of Caco-2 cells with LFA significantly reduced the adherence of 11168-O 25°C (P = 0.02), while no significant reduction was observed for 11168-O 37°C (P = 1.0) and 42°C (P = 0.21) (Figure 6B). Adherence assays using MAA and SNA as the neutralising lectin also revealed significant inhibition of 11168-O 25°C (P = 0.04 and 0.02, respectively), whereas no difference was observed with either MAA or SNA for 11168-O 37°C binding (Figure 6B). Taken together, this suggests that 11168, both the original and genome sequenced isolates, maintained under environmental conditions bind Neu5Ac independent of linkage, whereas 11168-GS 37°C predominantly binds α2-3-linked Neu5Ac.

**Figure 7:**
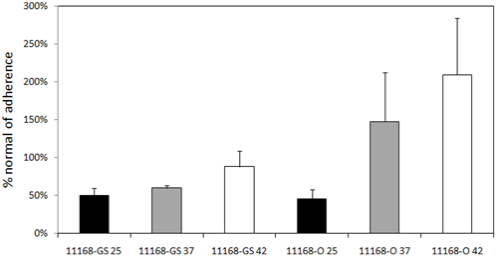
Relative adherence of *C. jejuni* 11168 to sialidase treated Caco-2 cells. Relative adherence of 11168-GS (left) and 11168-O (right) to sialidase treated Caco-2 cells with respect to normal adherence levels (25°C, black bar; 37°C, grey bar; and 42°C, white bar).

To further explore the significance of Neu5Ac in *C. jejuni* 11168 adherence to Caco-2 cells, the effect of Neu5Ac removal by sialidase treatment of Caco-2 cells was explored. Sialidase treatment of Caco-2 cells resulted in a 50% reduction in the adherence of both 11168-GS 25°C and 11168-O 25°C compared to non-sialidase treated controls (P = 0.03 and 0.02 respectively, Figure 7). Reduction in adherence to sialidase treated Caco-2 cells was also observed for 11168-GS grown at 37° (40% reduction) and 42°C (12% reduction); however, only 11168-GS 37°C was significantly different to untreated controls (P = 0.02, Figure 7). The opposite was observed for the adherence of 11168-O 37°C and 42°C to sialidase treated Caco-2 cells with increases of 47% and 109%, respectively. However, only the increase observed for 11168-O 42°C was significantly different to normal adherence observed for untreated Caco-2 cells (P = 0.04).

Lectin competition combined with sialidase treatment (summarised in Table 3) provided independent verification of the glycan array data, with inhibition of *C. jejuni* adherence observed using lectins specific for Gal-, Fuc-, Man- and Neu5Ac-containing glycans and differential effects of sialidase treatment being observed between the *C. jejuni* 11168 isolates.

**Table 3:** Summary of *C. jejuni* 11168-GS and 11168-O binding to glycan array.

Terminal glycan	11168-GS			11168-O		
	25°C	37°C	42°C	25°C	37°C	42°C
Man	++	+++	++	++	−	−
Le^a^	+	+++	+++	+	+++	+++
Le^b^	+	++	+++	+	++++	++++
Le^x^	+	+++	+++	+	+++	+++
Le^y^	+	++++	+++	+	++++	++++
α2-3Neu5Ac	++	++++	+	++	−	−
α2-6Neu5Ac	++	++++	+	++	−	−
Galβ1-3/4/6Glc/GlcNAc/Gal	−	++	++	+	+++	+++
Galα1-4/4Gal	−	++	++	+	+++	+++

−: Fluorescence <200 with no reduction in adherence caused by competing lectin.

+: Fluorescence <500 with no reduction in adherence caused by competing lectin.

++: Fluorescence <500 with significant reduction in adherence caused by competing lectin.

+++: Fluorescence >500<1000 with significant reduction in adherence caused by competing lectin.

++++: Fluorescence <1000 with significant reduction in adherence caused by competing lectin.

## Discussion

Bacterial pathogens and commensal organisms both utilise adherence to host cell surface glycans for colonisation and/or initiation of disease [2]. In this study we demonstrate that glycan array technology represents a powerful tool for the identification and characterisation of novel bacteria-glycoconjugate interactions. Specifically, we were able to interrogate the carbohydrate binding potential of two *C. jejuni* isolates, the genome sequenced and non-colonising *C. jejuni* 11168-GS isolate, and the original virulent and colonising *C. jejuni* 11168-O isolate. Significantly, a number of glycans were shown to specifically bind both isolates in a manner dependent on growth/maintenance conditions, including Man, Fuc, Gal and Neu5Ac, with each verified using a cell-based adherence assays (summarised in Table 3).

*C. jejuni* 11168-GS and 11168-O bound terminal Gal structures independent of linkage and the nature of the sub-terminal carbohydrate, with the most significant binding observed at growth conditions that mimic those of the potential hosts. The specificity of Gal-binding by *C. jejuni* was confirmed using cell adherence assays, where a significant decrease was observed for 11168-GS grown at 37°C and 11168-O grown at 42°C following pre-treatment of Caco-2 cells with the lectin ECA. It should be noted that ECA binds lactose with greater affinity than other terminal Gal-glycans. Therefore, glycan structures similar to the array glycans 1B, 1G and 1H would be preferentially neutralised by ECA over other terminal Gal structures (e.g. 1B, 1C, 1D).

Gal-binding lectins are common throughout nature and are involved in cell-cell and cell-matrix adhesion, cell signaling, toxin binding and cytokine release [2], [29], [30], [31], [32]. Bacterial pathogens produce a range of Gal-specific lectins that are used in various ways to interact with the host tissues. Several bacterial toxins target Gal on cell surfaces [2], [32], while other bacteria use Gal-binding lectins for adhesion, including enteroaggregative and enteropathogenic *E. coli* [33], [34], [35]. *E. coli* possesses an adhesin known as PapG, located on the flexible tip of P fimbriae, which binds Galα1-4Gal sequences present in the core of glycosphingolipids [36], [37]. *P. aeruginosa* expresses a soluble Gal-specific lectin, PA-IL, which facilitates adherence to the intestinal epithelium and also leads to increased permeability of the intestinal epithelial layer [4], [38]. The nature of the lectin, and the function of Gal-binding by *C. jejuni* identified here awaits elucidation. However, our investigations suggest that *C. jejuni* NCTC11168 possesses either one Gal- specific lectin with broad substrate specificity able to bind both α- and β- linked Gal, or two highly specific lectins able to bind Galα1-3/4Gal and Galβ- linked glycans separately that may be important for host (mammalian and avian) cell adherence. The finding that 11168-GS and 11168-O maintained at 25°C do not bind Galα1-3/4Gal sequences further suggests that this structure may be of particular importance in host adherence.

A previous study by Russell and Blake [39], showed that free Man was able to inhibit the adherence of *C. jejuni* to Caco-2 cells; however, the direct interaction of *C. jejuni* to Man has not previously been reported. Our present study has now demonstrated that *C. jejuni* 11168 is able to directly bind Man containing glycans in a specific manner. Interestingly, dramatic differences were observed between the ability of the GS and O 11168 isolates to bind Man. *C. jejuni* 11168-GS was able to bind a wide variety of Man structures independent of the growth/maintenance conditions. Conversely, *C. jejuni* 11168-O only bound Man after being maintained at 25°C under normal oxygen conditions. These results were confirmed by the ability of the Man-specific lectin ConA to inhibit the adherence of *C. jejuni* 11168 to Caco-2. Based on these findings it is therefore probable that *C. jejuni* 11168-O possesses a Man-binding lectin whose expression is dependent on the environmental temperature and/or atmospheric conditions encountered. Further, Man-recognition by *C. jejuni* 11168-O suggests that Man may be required for initial adherence to host tissues after environmental challenge, but may not be necessary for continued colonisation. Man is a ubiquitous component of *N*-glycan structures [40], used by many bacterial commensals and pathogens for adherence, such as uropathogenic *E. coli* that utilises the Man-specific lectin FimH located at the tip of type 1 fimbriae to bind host tissue [11], [41]. The vast majority of Man binding lectins identified in bacteria are, like FimH, associated with fimbriae or pili, extracellular adhesins that *C. jejuni* is not known to possess [42], [43]. While the Man-specific lectin of *C. jejuni* is unlikely to be of similar structure to previously identified fimbriae or pili lectins, the broad Man-binding specificity observed in *C. jejuni* is commonly observed in other bacterial species [41], [44].

It is not surprising that *C. jejuni* 11168 grown under mammalian and avian host-like conditions recognized multiple fucosylated glycans, including Le antigens. Fucosylated glycoproteins are frequently found in the intestines of humans and other animals [45], with Le antigens representing a common fucosylated glycan. Le antigens are classified as either type 1 (Le^a^ and Le^b^) or type 2 structures (Le^x^ and Le^y^) and are present, together with other fucosylated glycans, on mucins of the gastrointestinal tract [46]. Fucosylated glycans and free Fuc have been shown to interact with *C. jejuni* [24], [26]. In fact, free Fuc and Fuc associated with the O-glycans of mucins are considered *C. jejuni* chemotaxis agents [24]. It is therefore likely that *C. jejuni* proteins involved in chemotaxis towards Fuc may be, at least in part, responsible for the binding of *C. jejuni* to fucosylated structures present on our glycan array.

The ability of bacteria to bind to fucosylated host cell glycans has been linked to pathogenesis, with *P. aeruginosa* representing a well-characterised example. The *P. aeruginosa* Fuc-specific lectin (PA-IIL) has been structurally elucidated, and been shown to play a central role in *Pseudomonas* infection of immuno-compromised and cystic fibrosis patients [47]. PA-IIL facilitates the colonisation of the airways by adhering to the heavily fucosylated mucosal layer [47]. In *C. jejuni*, a mucosal pathogen, the importance of Fuc-containing glycoconjugates, particularly fucosylated glycoproteins found in human milk, was demonstrated by the ability of these structures to protect individuals against *C. jejuni* infection [48]. This protection against infection was attributed to the binding of *C. jejuni* to difucosylated terminal Le^b^ structures and 2'fucosyllactose present on human milk glycoproteins [26]. The importance of this interaction is highlighted by our observation that 11168-GS and 11168-O were able to bind at least one of the terminal Le^b^ or 2'fucosyllactose structures on our glycan array independent of the growth/maintenance conditions tested (Table 3, Figure 3A and 3B). Further, the complete inhibition of *C. jejuni* 11168-O 37°C adherence to Caco-2 cells by UEA-1 (terminal α1-2 Fuc binding lectin) confirmed the binding specificity of *C. jejuni* to terminal monofucosylated structures such as 2' fucosyllactose. It should be noted that *C. jejuni* 11168-O grown under mammalian host-like conditions was a poor binder of other non-Fuc capped glycans. However, the binding of lectins to a specific site on a cell surface glycoprotein may also result in the occlusion of other sites used by adhering bacteria, leading to a further decrease in binding to host cells.

The differential binding of fucosylated structures by *C. jejuni* grown or maintained under different conditions was also demonstrated by both the glycan array and UEA-1 pre-treatment of Caco-2 cells, with the most striking differences observed for both 11168-GS and 11168-O maintained at 25°C. Little or no binding was seen for both Le^a^ and Le^x^ under environmental conditions, with many other fucosylated structures showing reduced binding when compared to bacteria grown under host-like conditions. UEA-1 pre-treatment of Caco-2 cells had no effect on the level of adherence of environmentally maintained 11168. These data suggest that environmentally maintained *C. jejuni* 11168 is a poor binder of terminally α1-2 fucosylated structures compared to the same bacteria grown under host-like conditions. These results not only highlight the importance of host glycan fucosylation for *C. jejuni* adherence to intestinal cells, but also that *C. jejuni* grown under host like conditions is likely to express a broad specificity Fuc-binding lectin that mediates this interaction.

Much like Man-recognition, binding to sialylated glycans was observed for *C. jejuni* 11168-GS regardless of growth/maintenance conditions and for 11168-O 25°C, with little or no binding observed for 11168-O when grown under conditions mimicking mammalian or avian host (Table 3, Figure 4A and 4B). Significantly, these differences between *C. jejuni* 11168-GS and 11168-O are not due to a modification of glycans by the action of a recently described *C. jejuni* sialyltransferase/sialidase activity [28], with no significant differences in Neu5Ac binding observed in the presence of sialyltransferase or sialidase inhibitors.

As proposed for Man-binding, Neu5Ac may be important for the initial contact between environmental *C. jejuni* and host tissues. Neu5Ac is another highly abundant carbohydrate on cell surface glycoconjugates, representing a common receptor for lectins expressed by many pathogenic bacteria species and viruses [49]. Cell surface mucins are typically highly sialylated, only becoming fucosylated in disease states such as cystic fibrosis where there is large turnover of cell surface components, including cell surface mucins [47], [50]. The binding of *C. jejuni* to Neu5Ac-containing glycans present on our array was found not to be dependent on Neu5Ac-linkage or the nature of the sub-terminal sugar, regardless of the growth conditions tested and this was confirmed by LFA adhesion neutralisation assays. However, analyses using the Neu5Ac-linkage specific lectins SNA and MAA revealed selective specificity for α2-3-linked Neu5Ac, but only for *C. jejuni* 11168-GS grown at 37°C (Figure 5A).

Sialidase treatment of Caco-2 cells further verified the role of Neu5Ac in the adherence of *C. jejuni* 11168 maintained under environmental conditions with significant reduction in adherence observed for 11168-GS 25°C and 11168-O 25°C. A significant reduction in the adherence of 11168-GS 37°C was also observed verifying the array and Neu5Ac-binding lectin competition data. Sialidase treatment of Caco-2 cells also affected adherence of 11168-O grown under host-like conditions; however, unlike 11168-GS an increase in adherence was observed. Caco-2 cells have been shown to express α2-3Neu5Ac, α2-6Neu5Ac, α1-2Fuc and Gal, with terminal Neu5Ac structures consistently being reported in all studies [51], [52], [53], [54], [55]. Treatment with sialidase, thus removing terminal Neu5Ac residues, would therefore unmask sub-terminal structures such as Le^x^, lactose and neo-lactose [51], [52], [53], [55] enabling these glycans to be bound by *C. jejuni*. The observed increase in adherence of *C. jejuni* 11168-O 37°C and 42°C is therefore likely to be due to an increase in the number of these newly liberated glycans on the surface of Caco-2 cells following sialidase treatment. This is supported by the glycan array analysis that showed that Le^x^, lactose and neo-lactose were strongly bound by 11168-O grown under host like conditions (Table 3). These results indicate that 11168-O grown under host-like conditions is more likely to adhere to niches of the gastrointestinal tract with lower levels of sialylated glycoconjugates in comparison to either 11168-GS or environmentally challenged 11168-O.

Throughout this study both isolates of *C. jejuni* 11168 bound type-1 (Galβ1-3GlcNAc, Le^a^ and Le^b^) and type-2 (Galβ1-4GlcNAc, Le^x^ and Le^y^) structures present on the array. This is of interest as type-2 glycans are ubiquitously distributed throughout tissues of the body, while type-1 glycans are expressed only within certain tissues including the digestive tract and pancreatic tissues [56]. The binding of type-1 glycans is to be expected as *C. jejuni* primarily infects/colonises the gastrointestinal tract of animals; however, in human disease *C. jejuni* is known to also cause systemic infections including meningitis and urinary tract infections. The ability of *C. jejuni* to bind type-2 glycans may be an important virulence factor in systemic *C. jejuni* infections.

With the exception of fucosylated structures such as Le^b^, very little was previously known about the glycans recognised by *C. jejuni*. Our study has now identified a number of additional carbohydrate structures commonly found on the mucins of the gastrointestinal tract that are bound by both the original *C. jejuni* NCTC11168 (11168-O) and genome sequenced (11168-GS) isolates. Glycan array and cell adhesion assays indicate that these newly identified glycan-bacteria interactions are important for adhesion and possibly colonisation of host tissues during both infection and commensal colonisation.

Differential binding between growth/maintenance conditions of 11168-O highlights an apparent adaptation of glycan binding potential from environmental to host-like conditions, with Gal/Fuc binding being associated with host-like conditions and Man/Neu5Ac being associated with environmental conditions (summarised in Table 3). This indicates that *C. jejuni* 11168-O has a preference for gastrointestinal niches within host tissues, with terminal Gal/Fuc being preferentially bound over Man/Neu5Ac.

The most striking difference observed through our analyses was that the Man/Neu5Ac binding specificity of the *C. jejuni* 11168-O isolate varied significantly according to growth conditions, whereas this was not the case for the *C. jejuni* 11168-GS isolate. Much like 11168-O, *C. jejuni* 11168-GS grown under host conditions was observed to have increased binding to Gal/Fuc compared to environmentally maintained 11168-GS; however, 11168-GS grown under host like conditions still bound Man/Neu5Ac (Table 3). Therefore, given the data presented here, we postulate that the binding of Man/Neu5Ac may provide the initial interactions important for colonisation following environmental exposure. These interactions occur prior to bacteria adaptation through the expression of factors necessary for survival and growth in the avian and mammalian host. Presumably, changes in carbohydrate specificity from Man/Neu5Ac to Fuc/Gal through the expression of alternate lectins may then allow *C. jejuni* to adhere and colonise its preferred gastrointestinal niche.

Differential binding of glycans reported here, is likely to be one of several contributing factors to the differences in colonisation potential reported for the two *C. jejuni* 11168 isolates. [21]. Other factors contributing to the apparent lack of adaptation of *C. jejuni* 11168-GS to host-like conditions need to be considered, such as reduced motility of 11168-GS compared to 11168-O, and differences in the expression of genes involved in respiration (modulating *C. jejuni's* response to oxygen levels) and metabolism (modulating *C. jejuni's* response to temperature fluctuations) [21]. This report highlights the importance of further and detailed exploration of glycan-lectin interactions in the initiation and maintenance of *C. jejuni* colonisation and infection.

## Materials and Methods

### Preparation of SuperAmine Glass Slides

Propylamino-glass slides (SuperAmine2 glass slides, ArrayIt Microarray Technologies) were functionalised with a polyethylene glycol spacer moiety as previously described [57]. Glass slides were incubated for at least 18 h in a solution of Fmoc-8-amino-3,6-dioxaoctanoic acid (10 mM), PyBOP (10 mM), diisopropylethylamine (20 mM) in dry DMF at room temperature in an anhydrous environment. After fuctionalization the slides were rinsed in DMF, the Fmoc protecting group removed by incubating the slides in piperidine (10% v/v) in DMF for 30 min, and the slides were subsequently rinsed in DMF. The slides were then activated with isocyanate functionality by incubation in a DMF solution of 1,6-diisocyanatohexane (10% v/v) for 30 min at room temperature, rinsed once in DMF, once in THF, and centrifuged dry (1000 rpm for 2 min). The slides were stored under vacuum in desiccator for up to 1 week prior to use.

### Preparation of Glycosylamines

Glycans were purchased from Dextra Laboratories (Reading, UK) and Glycoseparations (Moscow, Russia), and functionalised with an amine group using a modification of a previously described methodology by Vetter and Gallop [58]. One to five milligrams of each glycan and ammonium carbonate (1.3 equivalents) were dissolved in ∼25% ammonium hydroxide (500 µL) in a sealed glass vial and incubated at 50°C for 3 days. The vials were unsealed, allowed to remain at 50°C for 2 h to dissipate excess ammonia and carbon dioxide, frozen and concentrated by lyophilisation. The glycosylamines were subsequently dissolved in anhydrous DMSO (100 µL), diluted further with an equal volume of anhydrous DMF and stored in sealed vials at 4°C until required.

### Preparation of Glycan Arrays

Arrays were printed by the robotic non-contact dispensing (Piezorray Non-Contact Dispensing System, PerkinElmer) of 1500 pL of 10 mM glycosylamines in DMSO/DMF (1∶1) onto isocyanate functionalised glass slides in replicates of four. Two identical sub-arrays were printed per slide with each sub-array comprising 352 spots (80 glycans and 8 controls in replicates of 4). Once the arrays had been printed they were placed in a vacuum desiccator fitted with a gas tap, which was evacuated under high vacuum. The vacuum was sealed off and a round-bottomed flask containing pyridine (∼1 mL) attached via hosing and the desiccator was re-equilibrated with catalytic pyridine vapour for at least 8 h. The slides were then neutralised by incubating them in ethylene glycol (1 M) and pyridine (1% v/v) in DMF for 30 min at room temperature. The glycan array slides were rinsed in DMF, then ethanol and centrifuged dry (900 rpm for 2 min), at which stage they were ready for binding assays.

### Bacterial Growth Conditions

*C. jejuni* strain 11168 original (11168-O; Skirrow), obtained from Prof Diane Newell (Veterinary Laboratory Agency, London, UK), and genome sequenced strain (11168-GS; NCTC) were grown at either 37°C or 42°C for 36 h or 20 h, respectively (see Table 1), on Columbia agar supplemented with 5% defibrinated horse blood in microaerobic conditions (10% CO_2_, 5% O_2_ in N_2_). For the maintenance of 11168-O and 11168-GS under environmental conditions bacteria were first grown at 42°C for 20 h prior to being harvested in 10 mL of sterile fresh pond water and incubated at 25°C as described in Table 1. Gram-staining was performed as per standard procedures, and was used to assess the level of coccoid bacteria present following growth/maintenance at the conditions stated in Table 1. Only *C. jejuni* maintained under environmental conditions were used in further experiments when bacteria were found to be coccoid.

### Labelling of *Campylobacter jejuni*

*C. jejuni* were harvested from agar plates using 1 mL of pre-warmed Brucella broth and pelleted by centrifugation at 13,000 rpm for 1 min. The pellet was washed 5 times with pre-warmed phosphate buffered saline pH 7.4 (PBS) to remove residual broth and agar, and diluted in PBS to 10^6^ bacteria/ mL. To label 10^6^ bacteria, 1 mL of diluted *C. jejuni* was pelleted by centrifugation at 13,000 rpm for 2 min and resuspended in 500 µL of 5 µM Carboxyfluorescein diacetate, succinimidyl ester (CFDA-SE, Molecular Probes) prepared in PBS and the labelling was performed using a modified version of that previously described [59]. Bacteria were then incubated protected from light either at room temperature for 90 min or at 37°C or 42°C for 30 min. Excess dye was removed by eight consecutive washes with 1 mL of PBS, with the final pellet being resuspended in 1 mL of PBS. Initial testing of CFDA-SE labelling was analysed with fluorescence activated cell sorting (FACS Calibur BD Scientific) with greater than 99% labelling observed. The extent of CFDA-labelling was routinely checked by fluorescence microscopy, with only bacterial samples observed to be greater than 90% labelling being used in subsequent experiments.

### Binding Assays to Glycan Array

Each of the two sub-arrays printed per slide were isolated with a 1.7×2.8 cm (125 µL) Gene Frame (Abgene), into which 125 µL of CFDA-labelled bacteria in PBS was added. Studies of the effects of sialyltransferase and sialidase inhibitors were preformed in the presence of CMP, Neu5Ac2en and 4-guanidino-Neu5Ac2en (Zanamivir) at a concentration of 10 mM in PBS. Each sub-array was covered with a Gene Frame coverslip (Abgene) and incubated in a humidified chamber at 37°C for 30 min. Slides were washed with 40 mL of pre-warmed PBS flushed across the array with a transfer pipette. Duplicate arrays were performed using separate cultures of the bacterial strains under investigation.

### Image Acquisition and Data Processing

Fluorescence intensities of array spots were measured using the ProScanArray Microarray 4-Laser Scanner (PerkinElmer) using the Blue Argon 488 excitation laser set to the FITC setting (494 excitation and 518 emission). Image analysis was carried our using the ProScanArray imaging software ScanArray Express (PerkinElmer). Microsoft Excel was used to further process the data and to perform statistical analysis (Independent sample T test).

### Caco-2 Cell Adherence and Lectin Neutralisation Assays

Adherence assays were performed using Caco-2 human intestinal cell lines. Cells were seeded at 10^5^ cells/well in a 96-well cell culture plates in minimal essential medium (MEM) for 48–72 h prior to bacterial challenge. Caco-2 cells were monitored prior to the assay to ensure a confluent monolayer of cells and that the cells had formed tight junctions. Sialidase pre-treatment of cells was carried out using 50 mU of *Vibrio cholerae* Sialidase (Sigma-Aldrich) for 2 h at 37°C in 5%CO_2_. Cells were pre-treated with the lectins UEA-1 (Sigma), ConA, ECA, MAA, SNA and LFA (EY Laboratories, Inc.) for 1 h, and subsequently washed 3 times with PBS. *C. jejuni* 11168, grown as described above, was added at a concentration of 5×10^6^ bacteria per well (viable counts were performed to verify the inoculums). Bacterial adherence was allowed to proceed for 1 h at 37°C under microaerobic conditions, with cells being subsequently washed three times with PBS. Caco-2 cells were then lysed with 0.1% Triton-X 100 in PBS and adherent bacteria enumerated by viable counts following plating onto Columbia agar with 5% horse blood and grown at 42°C for 36 h. All experiments were repeated a minimum of 3 times. Statistical analysis (independent sample Ttest) of viable bacterial counts was performed with Microsoft Excel.
